# Advantages of Fluoroscopy for Accidental Ingestion of Multiple Magnets

**DOI:** 10.1155/2023/1512514

**Published:** 2023-03-01

**Authors:** Toshihiko Kakiuchi, Tetsuya Nosho

**Affiliations:** Department of Pediatrics, Faculty of Medicine, Saga University, Saga, Japan

## Abstract

Foreign body ingestion is one of the common problems among children. A three-year-old girl presented to the emergency department 2 h after ingesting three small disk-type neodymium magnets. She had no gastrointestinal symptoms. Abdominal radiography revealed a foreign body in the left upper quadrant, which was the three circular magnets. Fluoroscopy was performed. She was placed in the right lateral decubitus position for 3 min and slowly returned to the supine position. Abdominal fluoroscopy revealed a foreign body migrating near the middle of the spine. Similarly, when she was placed in the left lateral decubitus position for 3 min and slowly returned to the supine position, the foreign body migrated to the outermost part of her left upper quadrant. Thus, we anticipated that the magnets could be excreted spontaneously. The patient did not develop any gastrointestinal symptoms after returning home, and two days later, three overlapping magnets were finally found in her stool. During accidental ingestion of single or multiple desk-type neodymium magnets, endoscopic removal may not be necessary if they are mobile and clump together in the stomach. Regarding the ingestion of multiple desk-type neodymium magnets, fluoroscopy is useful to determine the extent of endoscopic intervention.

## 1. Introduction

Foreign body ingestion is one of the common problems among children. There is the greatest tendency for children between the ages of six months and six years to have problems after placing objects in their mouths. These events can cause serious complications [1]. Ingestion of magnets is by no means a new occurrence in children [2], and cautions about the increased risk of injury with ingestion of multiple magnets have been in existence for many years [3].

## 2. Case Report

**Figure 1:**
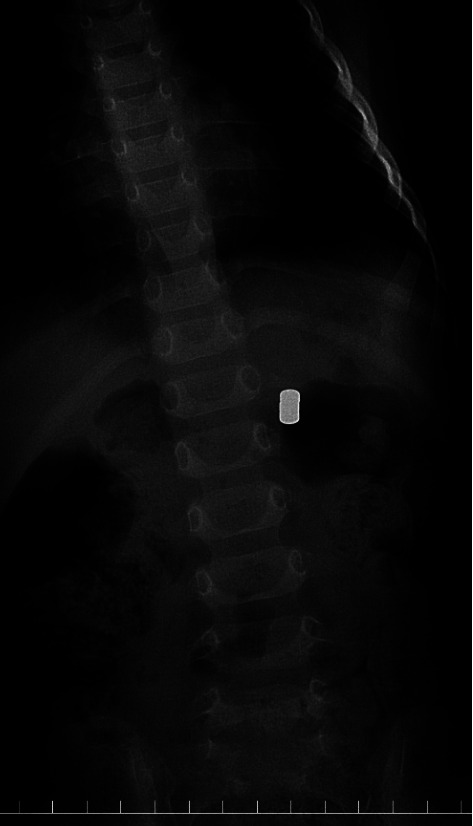
Abdominal radiography revealed a foreign body in the left upper quadrant, which were the three circular magnets.

**Figure 2:**
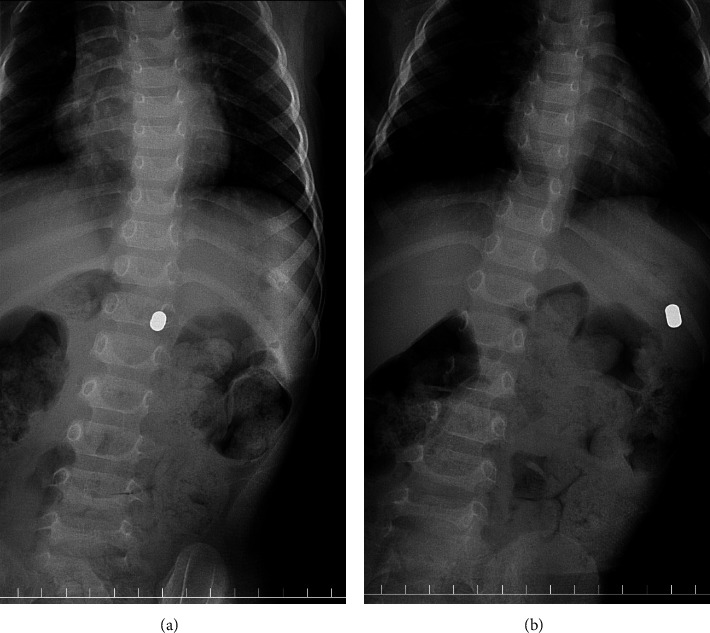
Abdominal fluoroscopy findings. (a) The patient was placed in right lateral decubitus position for 3 min and slowly returned to supine position. Abdominal fluoroscopy indicated the magnets were migrating near the middle of the spine. (b) When she was placed in left lateral decubitus position for 3 min and slowly returned to supine position, fluoroscopy revealed that the magnets migrated to the outermost part of her left upper quadrant.

A three-year-old girl presented to the emergency department 2 h after ingesting three small disk-type neodymium magnets (5 mm diameter × 3 mm thickness). Her body weight was 13.5 kg. She had no gastrointestinal symptoms. Abdominal radiography revealed a foreign body in the left upper quadrant, which was the three circular magnets (Figure 1). Since it had been 2 h after the accidental ingestion of the magnets, they had clumped together; hence, fluoroscopy was performed rather than endoscopy. She was placed in the right lateral decubitus position for 3 min and slowly returned to the supine position. Abdominal fluoroscopy revealed a foreign body migrating near the middle of the spine (Figure 2(a)). Similarly, when she was placed in the left lateral decubitus position for 3 min and slowly returned to the supine position, the foreign body migrated to the outermost part of her left upper quadrant (Figure 2(b)). Thus, we anticipated that the magnets could be excreted spontaneously. Since these were present in the stomach as a single mass, the overlapping magnets did not pinch the digestive tract, and the total length of the overlapping magnets was less than 1 cm. The patient did not develop any gastrointestinal symptoms after returning home, and two days later, three overlapping magnets were finally found in her stool.

## 3. Discussion

The ingestion of strong magnets can lead to life-threatening intestinal injuries, permanent disability, or death [4]. The ingestion of a neodymium magnet is unlikely to cause considerable damage; however, if multiple magnets are ingested or if a magnet is swallowed along with a metal object, they could be strong enough to attract each other along the intestinal walls and cause considerable damage [5]. The North American Society for Pediatric Gastroenterology, Hepatology & Nutrition guideline recommends that it is prudent to remove the magnet(s) endoscopically if possible, particularly if multiple magnets are in a location that is accessible by endoscopy [3].

In this case, although multiple desk-type magnets were ingested, they were excreted spontaneously. Removal of multiple desk-type magnets by invasive endoscopy may be avoided if the following conditions are met: the magnets are present in the stomach, the time from their accidental ingestion is short, multiple magnets are stuck together to form a single mass, and the mass of magnets can move around in the stomach. Fluoroscopy is useful to confirm this. On the other hand, in cases that do not meet these conditions, we believe that early endoscopic intervention should be prioritized according to the guidelines. Similarly, in facilities where fluoroscopy cannot be used, endoscopic intervention should be performed to remove multiple magnets. Moreover, if a follow up without an invasive method is adopted, it will be necessary to follow up with multiple radiography in outpatient and hospital settings [6]; however, this demand places a burden on patients and their families. Therefore, it is beneficial to confirm the mobility of the magnets during the initial fluoroscopy. However, this suggestion does not apply to magnetic beads because there is a risk for adjacent magnets to stick to each other across the intestinal tract depending on the length of the beads until they are excreted through the anus [7].

## Data Availability

The data used to support the findings of this study are available from the author upon request.
